# GeCoViz: genomic context visualisation of prokaryotic genes from a functional and evolutionary perspective

**DOI:** 10.1093/nar/gkac367

**Published:** 2022-05-26

**Authors:** Jorge Botas, Álvaro Rodríguez del Río, Joaquín Giner-Lamia, Jaime Huerta-Cepas

**Affiliations:** Centro de Biotecnología y Genómica de Plantas, Universidad Politécnica de Madrid (UPM) - Instituto Nacional de Investigación y Tecnología Agraria y Alimentaria (INIA-CSIC), Campus de Montegancedo-UPM, Madrid, 28223, Spain; Centro de Biotecnología y Genómica de Plantas, Universidad Politécnica de Madrid (UPM) - Instituto Nacional de Investigación y Tecnología Agraria y Alimentaria (INIA-CSIC), Campus de Montegancedo-UPM, Madrid, 28223, Spain; Centro de Biotecnología y Genómica de Plantas, Universidad Politécnica de Madrid (UPM) - Instituto Nacional de Investigación y Tecnología Agraria y Alimentaria (INIA-CSIC), Campus de Montegancedo-UPM, Madrid, 28223, Spain; Departamento de Biotecnología-Biología Vegetal, Escuela Técnica Superior de Ingeniería Agronómica, Alimentaria y de Biosistemas, Universidad Politécnica de Madrid (UPM), Madrid, 28040, Spain; Centro de Biotecnología y Genómica de Plantas, Universidad Politécnica de Madrid (UPM) - Instituto Nacional de Investigación y Tecnología Agraria y Alimentaria (INIA-CSIC), Campus de Montegancedo-UPM, Madrid, 28223, Spain

## Abstract

Synteny conservation analysis is a well-established methodology to investigate the potential functional role of unknown prokaryotic genes. However, bioinformatic tools to reconstruct and visualise genomic contexts usually depend on slow computations, are restricted to narrow taxonomic ranges, and/or do not allow for the functional and interactive exploration of neighbouring genes across different species. Here, we present GeCoViz, an online resource built upon 12 221 reference prokaryotic genomes that provides fast and interactive visualisation of custom genomic regions anchored by any target gene, which can be sought by either name, orthologous group (KEGGs, eggNOGs), protein domain (PFAM) or sequence. To facilitate functional and evolutionary interpretation, GeCoViz allows to customise the taxonomic scope of each analysis and provides comprehensive annotations of the neighbouring genes. Interactive visualisation options include, among others, the scaled representations of gene lengths and genomic distances, and on the fly calculation of synteny conservation of neighbouring genes, which can be highlighted based on custom thresholds. The resulting plots can be downloaded as high-quality images for publishing purposes. Overall, GeCoViz offers an easy-to-use, comprehensive, fast and interactive web-based tool for investigating the genomic context of prokaryotic genes, and is freely available at https://gecoviz.cgmlab.org

## Graphical Abstract

**Graphical Abstract:**
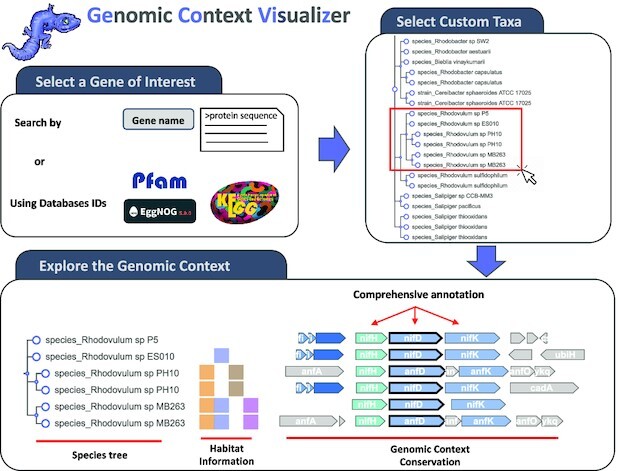
GeCoViz is an online resource providing fast and interactive visualisation of genomic context conservation of prokaryotic genes grouped by orthologous group or PFAM domains.

## INTRODUCTION

Bacterial and archaeal microorganisms possess packed genomes where functionally related genes, or those physically interacting, tend to cluster together (1,2), sharing regulatory mechanisms (3) and occasionally leading to gene fusion events (4). Thus, genomic context analysis has been extensively applied to predict the putative functional role of unknown genes (5–7). To obtain reliable predictions, comparative genomics methods use synteny conservation across multiple species as a strong indication of functional relationship (2,8,9). This approach has been proven useful in predicting protein-protein interactions (10), discovering novel functional roles (11), finding orphan enzymes (12) and characterising unknown metagenomics sequences (13).

The invaluable information derived from synteny conservation analysis has led to the development of numerous bioinformatic tools to automate the reconstruction of the genomic context of specific genes across multiple genomes (14,15) and explore it in a visual manner. Most notably, STRING uses genomic context conservation to predict protein-protein interactions (16), allowing also to display the neighbourhood of specific genes across different branches of the tree of life as a schematic representation. WebFlaGs (17) and TREND (18) can be used to generate static images representing the genomic context of custom genes based on previous computations of homologous sequences. GeConT2 (19) focuses on providing online searches on reference genomes, while GeneSpy (20) can be used to generate custom plots based on local computations. However, despite the unquestionable value of previous software, tools are still missing that provide fast and interactive exploration of genomic context conservation of prokaryotic genes while keeping a comprehensive phylogenetic and functional scope.

Here, we present GeCoViz, a highly interactive web application that aims at visualising genomic context conservation while offering a responsive and easy-to-use interface accessible to non-expert users. To provide fast searches, GeCoViz uses precomputed information on orthology assignments, phylogenetic information and functional annotations for over 42 million genes extracted from 12 221 reference prokaryotic genomes. Moreover, GeCoViz offers highly customizable searches, allowing users to easily adjust the phylogenetic scope of each analysis and select what functional annotations of neighbouring genes are used to highlight synteny conservation. Notably, GeCoViz uses eggNOG v5 (21) for its orthology assignments, enabling the exploration of thousands of hypothetical genes that are missing in other databases (COG, KEGG, PFAM) but still classified as orthologous groups of unknown function in eggNOG. This is particularly relevant for the characterization of unknown genes without experimentally validated homologs in any reference genome, which account for nearly one third of all the predicted genes in current databases.

## RESULTS

### Phylogenetic and functional scope

GeCoViz is built upon the reference set of genomes provided in proGenomes v2.0 (22), which includes 11 710 and 511 representative bacterial and archaeal species, respectively. In total, GeCoViz covers 42 542 377 protein coding genes, which were pinpointed to their location in their respective genomes. Comprehensive functional predictions and orthology assignments were computed de novo for all gene entries using eggNOG-mapper v2.1 (23). In total, 34 554 188 genes (81.2%) were mapped to at least one orthologous group in eggNOG v5, 21 671 011 (50.9%) were annotated to KEGG modules and pathways (24), and 32 215 373 to PFAM domains (25). Overall, we estimate that besides the core set of genes putatively assigned to known KEGG pathways or having known domains, GeCoViz enables the exploration of 5 390 779 highly hypothetical genes (no KEGG or known PFAM domains), spanning 1 304 714 eggNOG orthologous groups of unknown function.

### Hypothesis driven exploration of genomic context

GeCoViz allows users to look up the genomic context of any bacterial and archaeal gene, automatically estimating the conservation of their genomic neighbours along a customly selected set of species. Searches can be performed by using gene names, protein sequences or orthologous groups identifiers from either eggNOG v5 or KEGG databases as a query. When a single gene name or protein sequence is queried, GeCoViz uses precomputed orthology assignments to automatically identify equivalent genes (putative orthologs) along the different genomes selected. In addition, PFAM names can also be queried in order to explore the context of genes sharing the same protein domain. Since many hypothetical proteins are covered by eggNOG groups of unknown function, GeCoViz can be easily used to explore their genomic context and obtain hints on their possible functional role. Thus, GeCoViz allows users to perform hypothesis-driven searches to either inspect the functional context and synteny conservation of uncharacterized genes, or to explore differences in the genomic organisation of known molecular functions and pathways across a custom set of genomes and organisms (see example use cases).

### Interactive exploration of genomic neighbourhood

**Figure 1.**
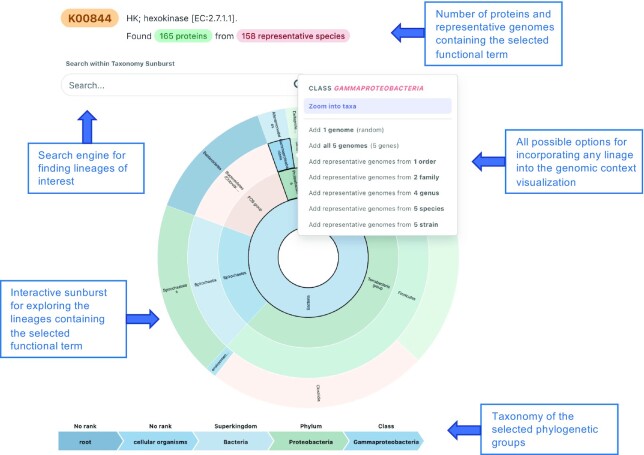
Taxonomic selector panel in GeCoViz. The taxonomic selector offers an interactive way of adjusting the phylogenetic scope of each analysis. The interface consists of a zoomable sunburst that presents the number of hits found (genomes with genes containing the functional term of interest) at each taxonomic level. Users can choose to add either all genomes under a given clade or a subset of representative genomes at different taxonomic resolutions.

GeCoViz offers a highly interactive and customizable exploration panel. When a new search is triggered, genomic context and synteny conservation is automatically shown for up to 100 homologous genes automatically selected from all major lineages in the prokaryotic phylogeny where the query term was found. Then, the taxonomic scope can be easily adjusted by means of the interactive sunburst chart available at the taxonomic control panel (Figure 1), allowing users to automatically add or remove representative species from custom clades, or manually select specific genomes.

**Figure 2.**
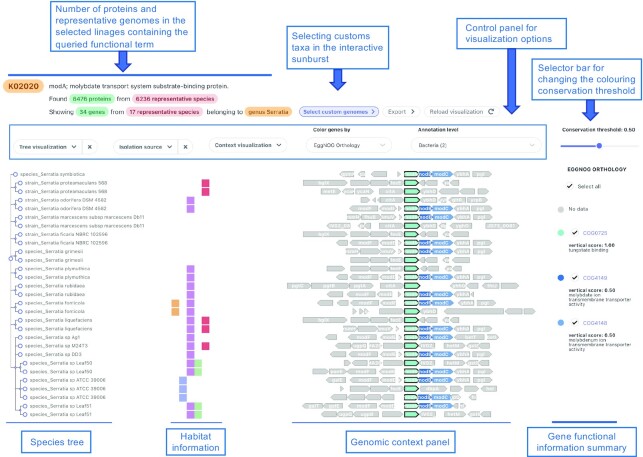
Genomic context panel in GeCoViz. The genomic context visualisation panel allows users to explore the genomic neighbourhood of target genes across different genomes, where phylogenetically conserved genes are coloured based on a custom VSC. When clicking on any gene, extensive functional information is shown, including eggNOG, KEGG, and Pfam annotations, allowing also to download individual sequences. By means of the context visualisation menu, users can increase or decrease the number of up- and down-stream neighbouring genes shown, choose between scaled or schematic representation of genes, and select the functional source used for colouring genes. Users can export their current view either as a high-resolution image (SVG and PDF) or as raw data (i.e. positional information of each gene in their respective genome).

Genes matching the original query—which are automatically grouped by either orthology, domain or metabolic pathway annotation—are vertically aligned in the genomic context panel and used as an anchoring point to display up- and down-stream loci for each genomic region (Figure 2). The genomic window size and graphical aspect of the genomic context representation can also be adjusted by the user. For instance, scaled gene lengths and genomic distances are displayed by default, but an alternative unscaled visualisation mode can be selected from the context visualisation menu. Moreover, a guiding tree sorting genomic regions by the NCBI Taxonomy, as well as additional habitat information for each species, can be optionally shown in the genomic context panel.

To facilitate the analysis of synteny conservation of particular genes across selected genomes, GeCoViz dynamically calculates a vertical conservation score (VCS) for each gene entry. The VCS can be estimated based on any of the annotations associated with the genes shown, such as the eggNOG orthologous groups restricted to custom taxonomic levels, KEGG orthologs and pathways, and PFAM domains. VCS is calculated as the percentage of genomes that contain the selected term (e.g. eggNOG, KEGG, PFAM), over the total number of genomes shown. Users can adjust the threshold of this simple score to highlight and colour genes that are more or less prevalent across the selected set of genomes, facilitating the identification of conserved patterns.

Moreover, users can interact with each gene entry by either clicking or hovering on its graphical representation. While gene clicking displays a window with detailed description of its location and annotated function, gene hovering immediately highlights other genes belonging to the same orthologous group across all the genomes. Besides, users can interact with the genomic context panel by: (i) hiding specific genomes by clicking on their respective tree nodes, (ii) showing their isolation source, (iii) expanding the size of the genomic window by increasing the number of up- and down-stream neighbouring genes shown and (iv) displaying genes in schematic format. Finally, users may download: (i) a table with the complete genomic context information including functional annotations, gene order and all the gene sequences and (ii) high quality images of any custom view of the genomic context analysis.

## EXAMPLE CASE STUDIES

### Predicting the functional role of hypothetical proteins

**Figure 3.**
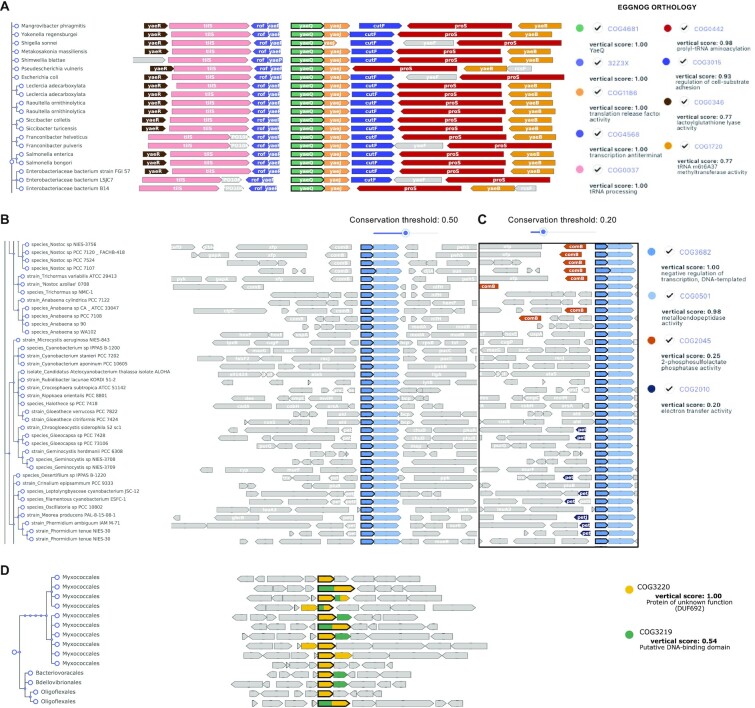
Use case examples with GeCoViz. (**A**) Genomic context of the hypothetical protein STM2039 (COG4681) from *Salmonella enterica* sv. Typhimurium LT2 obtained in GeCoViz. 55 representative enterobacteriaceae genomes are shown. Most conserved neighbouring genes around *STM2039* (*yaeQ*; green) encode for functions related to translation. (**B**) Genomic context of the transcriptional regulator *petR* (COG3682; blue) in a representative set of 38 cyanobacterial genomes reveals *petP* (COG0501; light blue) as the highly conserved attached gene (conservation threshold: 0.5). (**C**) By lowering the conservation threshold, GeCoViz allows to identificate the moderately conserved genes *comB* (COG2045; VCS 0.25) and *petJ* (COG2010; VCS 0.20). (**D**) Visualisation of the genomic context of COG3220 across representative species of 82 different bacterial orders. Genes belonging to COG3220 are usually next to COG3219 (putative DNA-binding domain) with VCS > 50%. In several orders, as it could be observed for Myxococcales and Oligoflexales, both genes are fused. For visualisation purposes in all the panels, some nodes were collapsed using the GeCoViz collapse node function, available when clicking on any node of the tree.

The *Salmonella enterica* sv. typhimurium LT2 coding gene *STM0239* (*yaeQ*) is currently annotated as a hypothetical protein in NCBI. Sequence search in GeCoViz assigns STM0239 to the orthologous group of unknown function COG4681 (eggNOG), which can be directly queried in GeCoViz. Exploration of COG4681 shows that *yaeQ* has 1959 orthologous widely distributed along the bacterial phylogeny and is embedded in a highly conserved region for all species under the enterobacteriaceae family (Figure 3A). Most of the conserved neighbouring genes of *yaeQ* are involved in tRNAs metabolism, and the gene itself is in an operon-like structure with *yaeJ*, which encodes for a translation release factor. This suggests that *yaeQ* might indeed be involved in translation processes, which supports previous works reporting that YaeQ has a role regulating the expression of virulence factors in both *Escherichia coli* and *Salmonella typhimurium* (26,27).

### Discovering novel genes associated with known pathways

Similarly, genomic context can provide valuable insights not only for functional but also for regulatory relationships between neighbouring genes. GeCoViz facilitates the discovery of putative target genes of known regulatory systems by means of the custom adjustment of taxonomic scopes and VCS thresholds. To illustrate this, we analysed the genomic context of PetRP, a recently described regulatory system involved in the plastocyanin (PC)/cytochrome c6 (C6) switch in cyanobacteria (28). Although the copper regulation of these two proteins in cyanobacteria was established 30 years ago (29), the regulatory system remained elusive until its recent identification by a genomic context approach (28). In this work, the role of PetR (Slr0240 *Synechocystis* sp. PCC 6803 protein), a homologous of the copper transcriptional regulator CopY, as a potential regulator of the PC/C6 switch was explored. Searching Slr0240 by protein sequence in GeCoViz assigned it to the eggNOG COG3682 orthologous group, a transcriptional negative regulator in bacteria. An initial visualisation of the genomic context of COG3682 in the cyanobacteria phylum reveals that *petR* (Slr0241 *Synechocystis* protein) is always attached to the metallopeptidase coding gene *petP* (eggNOG 1G0TE) (Figure 3B), with no other genes highly conserved in their context. However, by decreasing the VCS threshold to ∼20%, GeCoViz highlights two other neighbouring genes moderately conserved around *petRP*: *comB*, encoding for a 2-phosphosulfolactate phosphatase, and the experimentally validated target of PetR, *petJ* (C6; Figure 3C) (28).

### Identifying gene fusion events

Besides genome context conservation, GeCoViz also allows to easily spot eventual gene fusions. For instance, the exploration of genes of unknown function under the orthologous group COG3220 reveals that the target gene is tightly coupled to a putative DNA binding protein (COG3219; Figure 3D), occasionally leading to fusion events in several bacterial orders, reinforcing their functional relationship as a DNA interacting protein.

## IMPLEMENTATION DETAILS

GeCoViz uses MongoDB (https://www.mongodb.com/) for storing precomputed genomic data and Django on the server side (https://www.djangoproject.com/). The web frontend uses Vue.js (https://vuejs.org/) as a Javascript framework. Code for generating genomic context layouts hinges on the data visualisation library D3.js (https://d3js.org). Data flow and technical implementations are depicted in Supplementary Figure S1.

## DATA AVAILABILITY

GeCoViz is available at https://gecoviz.cgmlab.org.

## Supplementary Material

gkac367_Supplemental_File
