# Supplementary figures and images for: GeCoViz: genomic context visualisation of prokaryotic genes from a functional and evolutionary perspective
Source: Nucleic Acids Res. 2022 May 26;50(W1):W352–7. doi: 10.1093/nar/gkac367 (PMC9252766; doi:10.1093/nar/gkac367)

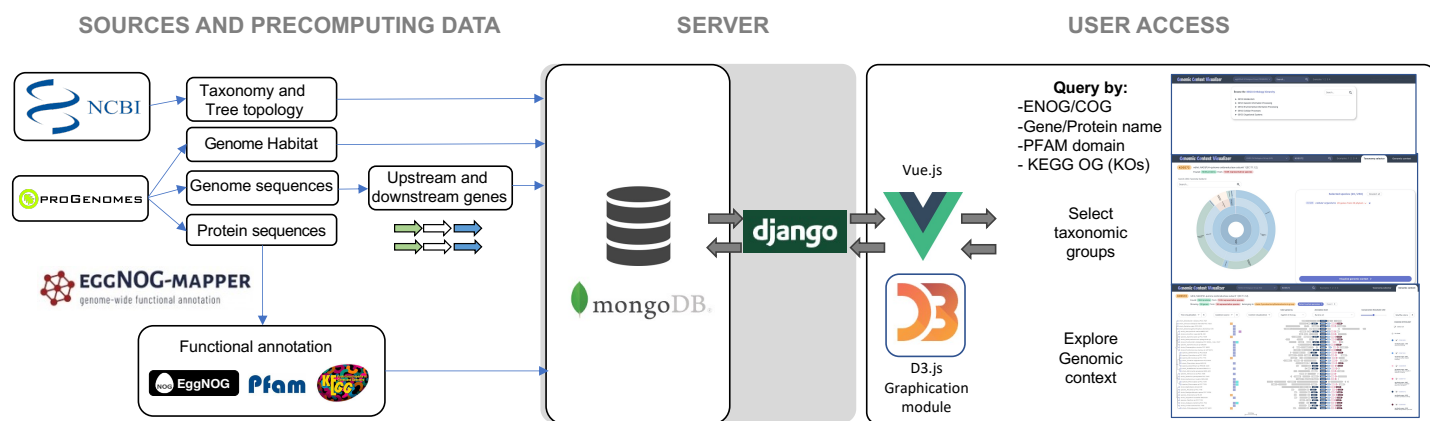

**Supplementary figure S1.** Data flow and technical implementation in GeCoViz.

Supplement: gkac367_Supplemental_File [file gkac367_supplemental_file.pdf]
